# Cross-referencing French hematology teams’ knowledge and perception of end-of-life situations: a national mixed-methods survey

**DOI:** 10.1186/s12904-025-01659-9

**Published:** 2025-01-31

**Authors:** Chloé Prod’homme, Côme Bommier, Laurène Fenwarth, Stephane Moreau, Alice Polomeni

**Affiliations:** 1 Univ. Lille, ULR 2694 METRICS, CHU Lille, Palliative care unit, Lille, F-59000 France; 2 Hôpital St Louis, Inserm U1153 ECSTRRA Team, Hémato-Oncologie DMU DIH, Paris, F-75010 France; 3 Univ. Lille, CNRS, Inserm, UMR9020-U1277 - CANTHER - Cancer Heterogeneity Plasticity and Resistance to Therapies, CHU Lille, Hematology Laboratory, Lille, F-59000 France; 4 CH Brive Hematology, Boulevard Verlhac 19032, Brive la Gaillarde, France; 5 Department of clinical hematology and cellular therapy, Hôpital Saint Antoine, Assistance Publique-Hôpitaux de Paris, Paris, France

**Keywords:** End-of-life care, Surveys, Hematology, Euthanasia

## Abstract

**Introduction:**

Haematology is a speciality frequently confronted with end-of-life situations, and teams will be concerned by the question of medical assistance in dying. The Ethics Commission of the French Society of Haematology has conducted a survey on the knowledge and perceptions of healthcare professionals regarding complex end-of-life situations.

**Methods:**

A cross-sectionalonline survey of hematology professionals in France. The comprehensive online questionnaire addressed respondents’ experience of complex end-of-life situations in hematology, based on 7 clinical vignettes. The survey contained 55 questions, 6 of which were open-ended. They were asked to give their opinion on whether it should be legalized. Justifications were then requested and analyzed by theme.

**Results:**

The survey was distributed to associations of hematology healthcare professionals (approximately 1,300 members). Overall, 182 healthcare professionals replied, including a third nurses and a third physicians. The average score for identifying complex situations was 7.1 out of 10 (IQR 5.7,8.6), with lesser knowledge of situations involving double effect, euthanasia and sedation for distress than of situations involving limiting or stopping treatment. Training in palliative care was the main driver of knowledge (*p* = 0.004), as well as being a physician (*p* < 0.001). We found that the opinions of healthcare professionals regarding the legalization of medical assistance in dying in France were diverse and well-founded.

**Conclusion:**

Hematology healthcare professionals had lesser knowledge of situations involving double effect, euthanasia and sedation for distress. Knowledge of specific situations impacts professionals’ opinion on legalization of medical assistance in dying.

**Supplementary Information:**

The online version contains supplementary material available at 10.1186/s12904-025-01659-9.

## What is already known on this topic

A societal debate on legalizing medical assistance in dying (MAID) began in 2023 in France. Haematology is a discipline frequently confronted with end-of-life situations, but collaboration with Pallative Care teams is difficult. It therefore seems foreseeable that French haematology teams will be particularly concerned by the question of MAID.

## What this study adds

Hematology healthcare professionals have lesser knowledge of situations involving double effect, euthanasia and sedation for distress. Knowledge of specific situations impacts professionals’ opinion on legalization of MAID.

## How this study might affect research, practice or policy

Training in palliative care is a major issue in haematology departments, which are faced with many complex end-of-life situations. Correctly identifying the different end-of-life care situations is an essential step to offering patients a high-quality palliative care, while strengthening their access to rights guaranteed by a changing legal framework.

## Background

Laws allowing patients who meet specific eligibility criteria to request medical assistance in dying (MAID) are increasingly being adopted in various countries [1–4]. MAID may refer to euthanasia, physician assisted dying or voluntary assisted dying, depending on the country. They may describe different situations such as the prescription of an oral lethal treatment that the patient administers himself, or the infusion by a physician of a lethal product at the patient’s request [5].This trend confronts healthcare professionals and facilities with complex ethical and clinical issues [6].

Around the world, several jurisdictions have taken a stand against MAID. For example, Switzerland decriminalises assisted suicide under certain conditions. Oregon has authorised MAID since 1994. The Netherlands has decriminalised euthanasia and physician-assisted suicide since 2001. Belgium has only decriminalised euthanasia since 2002. Canada has authorised euthanasia and assisted suicide since 2016, Australia since 2017 and Spain since 2021 [7].In France, the latest law of 2016 on patients’ rights at the end of life made it possible to access deep and continuous sedation until death in the event of refractory suffering at the end of life. A societal and parliamentary debate on legalizing MAID began in 2023, following the 139 public note by the National Consultative Committee of Ethics [8, 9]. In this notice, the experts raised the possibility of MAID because of shortcomings in current French legislation, subject to accelerated development of palliative care (PC) throughout the country.

Haematology is a discipline frequently confronted with end-of-life situations, where patients have considerable needs in terms of PC and overall management [10]. However, collaboration with PC teams is more difficult for haematologists than for oncologists treating patients with solid tumors [11].

Barriers to the integration of PC into haematology care are numerous and well described in the medical literature [12–14]. These include obstacles stemming from the cultural model of haematologists: on the one hand, their preference for an individual relationship right up to the end of the patient’s life, without referral to a third party team; on the other hand, their desire to preserve the patient’s hope by pursuing specific therapeutic proposals shortly before death [15–17]. It therefore seems foreseeable that haematology teams in France will be particularly concerned by the question of their patients’ access to MAID.

Indeed, there is a fear that this request may be a “default” solution for vulnerable patients and families with limited access to PC [18, 19]. Another apprehension concerns the risk of societal pressure in favor of MAID on the frailest people, who may be considered a “burden on society” because of their dependence, vulnerability and/or the seriousness of their illness. Yet this pressure would only exacerbate the existential suffering of patients at the end of life, that PC teams seek to alleviate, in particular by recognizing the value of others, whatever their situation [5].

The institutions and people involved in MAID seem to have adapted over time, following the introduction of new laws [20, 21]. For example, MAID is not only carried out in specialized or PC facilities [22–25]. This is why there is a huge need for training and support for all clinicians in all specialties, so that these new experiments can be used to improve thinking, practice and skills.

Differentiating and identifying end-of-life (EOL) situations is an essential foundation for ethical clinical practice. The role of palliative care has been to enrich the vocabulary and language of EOL care, in order to clarify prescribers’ intentions and better adjust management. For instance, sedative practices have long been confused with euthanasia, and thus remain controversial [26–28]. How much do hematology teams know about recognizing these EOL situations, at a time when legislation is likely to move towards legalizing MAID?

In order to provide haematology teams with a basis for ethical reflection on this issue, the Ethics Committee of the French Society of Haematology (SFH) aimed to investigate the understanding of Law-defined complex EOL situations and the way they are perceived among health care providers working in hematology in France.

## Methods

### Design

We constructed an cross-sectional online survey with a series of open-ended questions of hematology professionals in France. Our mixed-method survey study has been performed in accordance with the relevant guidelines [29, 30].

### Sample

The sample consisted of health professionals working or having worked in an inpatient hematology unit in France. They were informed about study through newsletters from the SFH (about 1000 members), the French Association of Residents in Hematology (about 250 members), the AFITCH-OR (French Association of Nurses working in cell therapy, hematology, oncology and radiotherapy )(about 70 members).

### Procedure

Participants were invited by email in February 2023 to participate in the survey anonymously. A reminder was sent 2 weeks later. After that, a visual information about the survey was displayed in all the rooms of the SFH national congress in March 2023 for 3 days. Participants were self-recruited after being informed (voluntary sampling). The survey was conducted on the internet. It was open from February 10th, 2023, to April 30th, 2023.

### Survey

The study-specific questionnaire was developed by physicians in haematology and PC (*n* = 8), based on clinical and educational experience. Clinical situations described in the survey were defined according to recommendations of French society of palliative care, French health authority and National Consultative Ethics Committee. After that, 10 professionals in hematology and members of the ethics committee of the SFH (psychologist, physicians and nurses) tested the questionnaire. It has been improved to make it easier for all healthcare professionals to understand and tested again by all the professionals (*n* = 18).

The comprehensive online questionnaire contained 55 questions and addressed respondents’ experience of complex EOL situations in hematology, i.e., treatment limitation and discontinuation, double-effect treatment, sedation for distress, deep and continuous sedation maintained until death, euthanasia and assisted suicide. Seven clinical vignettes described all these situations (euthanasia was described in two vignettes) (The English translation of the questionnaire is provided in the supplementary data S1). Firstly, respondents were asked to identify each situation (only one possible choice and one good answer), before describing its lawfulness and its routine practice. Then, if the situation was illegal in France, they were asked to give their opinion on whether it should be legalized and whether they would agree to carry out these acts. Justifications were then requested (open-ended answers). The questionnaire ended with the collection of socio-demographic data.

### Ethical considerations

The study was approved and promoted by the ethics committee of the SFH, which implied that individual informed written consent from participants was unnecessary. Answers were collected through the RedCap software. The information transmitted has been secured in compliance with French National Commission on Information and Liberty regulations.

### Quantitative analysis

Incomplete questionnaires were ruled out. Descriptive methods were used to present quantitative data. Since there was only one good answer by situation, the raw scores could range from 0 to 7, which were standardized using a linear transformation into scores ranging from 0 to 10. The overall knowledge was based on answers given to both “identification” and “lawfulness” questions, Respondents were divided into three categories, according to their overall knowledge standardized score: extensive overall knowledge if > 7.5/10, average overall knowledge if 5-7.5/10, limited overall knowledge if < 5/10. Results were analyzed according to overall knowledge category, age category, professional category, and to ethics or PC training. Respondents’ scores were compared between categories using Chi-square test of independence, Fisher’s exact test or Kruskal-Wallis test, depending on the nature of variables to be compared. Univariate analysis were conducted using generalized linear models and subsequent multivariate analysis using stepwise selection among candidate variables were derived to evaluate the effect of these variables on the knowledge score. We explicitly asked the sub-group of respondents who correctly recognized the different situations if they encountered these cases in their department. After that, opinions on legalization of euthanasia and assisted suicide were compared according to their knowledge of these very practices.

### Qualitative analysis

The purpose of the 6 open-ended questions (2 questions by clinical vignette corresponding to MAID practices) was to explore the arguments put forward by the participants to justify the positions they had taken on euthanasia and assisted suicide.

The thematic analysis was based on the guide proposed by Braun and Clarke [31]. All analyses were carried out by two authors with experience in qualitative research. The verbatims were first analysed by floating reading, which made it possible to read all the verbatims (CP, AP and LF). This step made it possible to identify the main themes contained in the verbatims (CP, AP and LF). Each verbatim was then freely coded by key words and key concepts by two reviewers who reread all the verbatims (CP and AP). The results were pooled. Any discrepancies in the coding of key words or concepts were resolved by discussion and consensus (CP, AP, LF, CB).

The categorical data were presented as numbers and percentages.

The thematic analysis of the responses to the questions relating to the justification for legalizing MAID added other sub-themes to the initial coding that had not been measured in terms of occurrences: they are illustrated by the verbatim statements deemed most significant (either those that occur most often or those that make it possible to clarify the data analysis) in order to illustrate the issue under study. The results of the thematic analysis were reviewed and validated by the various authors.

## Results

### Sample characteristics

Of the 182 respondents, 133 (73%) were women, 106 (58%) were aged 30–50, and 161 (88%) were working in a hematology unit at the time of the questionnaire. The most represented professional categories were tenured senior consultants (non-academics, 34%) and nurses (29%), and most of the respondents worked in a university hospital (tertiary care, 79%). Fifty-one (28%) participants declared having a training in medical ethics or PC. Respondents’ characteristics are provided in Table 1.

**Table 1:** Respondents’ characteristics. Statistics presented: n(%)

Characteristics	*n* = 182
Sex, female	133 (73%)
Age (years)	
*<30*	34 (19%)
*30–50*	106 (58%)
*51–60*	30 (16%)
*>60*	12 (7%)
Professional category	
*Resident*	22 (12%)
*Senior fellow*	9 (5%)
*Senior consultant (non-academic)*	62 (34%)
*Academic consultant*	12 (6%)
*Nurse*	52 (29%)
*Patient care assistant*	3 (2%)
*Psychologist*	7 (4%)
*Other*	4 (2%)
*No answer*	11 (6%)
Specialty of routine practice	
*Hematology*	161 (88%)
*Oncology*	5 (3%)
*Paliative care*	6 (3%)
*General practice*	2 (1%)
*Other*	7 (4%)
Facility of routine practice	
*University hospital (tertiary care)*	143 (79%)
*Community hospital (secondary care)*	22 (12%)
*Comprehensive cancer center*	7 (4%)
*Private clinic*	6 (3%)
*Other*	4 (2%)
Country of routine practice	
*France*	179 (98%)
*Other*	3 (2%)
Training in ethics or palliative care	51 (28%)

### Knowledge of end-of-life situations

The median score of situation identification was 7.1 (IQR 5.7,8.6) and the median legal knowledge score was 9.3 (IQR 8.6, 10.0), which translated into a median overall knowledge score of 7.1 (IQR 5.7,8.6). The three situations with the lower rates of knowledge were “double-effect treatment” (46%), “euthanasia” (46%), and “sedation for distress” (50%). The best-known situation was “treatment limitation and discontinuation” (92%).

Expectedly, the median overall knowledge score ranged from 7.1 (IQR 5.7,8.6) in respondents with limited knowledge to 8.6 (8.6, 10.0) in those with extensive knowledge. Extensive overall knowledge was mostly seen in youngsters (50% of < 30-year-olds, 77% of residents) and in respondents with a training in ethics or PC (45%). Specific knowledge of each situation was significantly different for each situation (*p* < 0.001 in each situation). Age below 50 was associated with a higher capacity to identify situations (*p* = 0.012), and with a better knowledge on “double-effect treatment” (65% in < 30-year-olds vs. 45% in respondents aged 30–50 vs. 31% in respondents aged 50+, *p* = 0.034), see Table 2. In univariate and multivariate analysis, the baseline variables associated with a higher knowledge score were being a physician (*p* < 0.001) and having trained in Ethics/PC (*p* = 0.004). Details are provided in supplementary data S2.

Training in ethics or PC was associated with a better overall knowledge and a better knowledge on “treatment limitation and discontinuation” (*p* < 0.001) and “double-effect treatment” (*p* = 0.039, see supplementary data S3).

**Table 2:** Knowledge on EOL situations according to age category. Statistics presented: median (IQR: interquartile range); n(%). Statistical tests performed: Kruskall-Wallis test; chi-square test, Fisher’s exact test

	All	Age (years)				*p*-value
		< 30	30–50	51–60	> 60	
		*n* = 34	*n* = 106	*n* = 30	*n* = 12	
Overall knowledge	7.1 (5.7,8.6)	7.9 (5.7, 9.6)	7.1 (5.7, 8.6)	5.7 (4.3, 7.1)	7.1 (5.4, 7.5)	0.13
*Extensive*	59 (32%)	17 (50%)	33 (31%)	6 (20%)	3 (25%)	
*Average*	82 (45%)	9 (26%)	53 (50%)	14 (47%)	6 (50%)	
*Limited*	41 (23%)	8 (24%)	20 (19%)	10 (33%)	3 (25%)	
Identification of situations (/10)	7.1 (5.7,8.6)	8.6 (7.1,10.0)	8.6 (8.6,10.0)	5.7 (5.7,7.1)	7.1 (5.4,7.5)	0.012
Legal knowledge (/10)	9.3 (8.6,10.0)	10.0 (8.6,10.0)	7.1 (5.7,8.6)	10 (7.1, 10.0)	10.0 (9.6,10.0)	0.4
Specific knowledge						
*Double-effect treatment*	83 (46%)	22 (65%)	48 (45%)	9 (30%)	4 (33%)	0.034
*Treatment limitation and discontinuation*	168 (92%)	31 (91%)	98 (92%)	28 (93%)	11 (92%)	0.9
*Sedation for distress*	92 (51%)	16 (47%)	58 (55%)	14 (47%)	4 (33%)	0.5
*Deep and continuous sedation maintained until death*	143 (79%)	25 (74%)	86 (81%)	21 (70%)	11 (92%)	0.4
*Assisted suicide*	131 (72%)	27 (79%)	74 (70%)	19 (63%)	11 (92%)	0.2
*Euthanasia*	83 (46%)	21 (62%)	48 (45%)	10 (33%)	4 (33%)	0.11

### Perception of legal and illegal practices

Among the six discussed practices in this study, four were legal (treatment limitation and discontinuation, double-effect treatment, sedation for distress, and deep and continuous sedation maintained until death) and two were illegal (euthanasia and assisted suicide) in France at the time of the survey. In this analysis, we only report the responses of respondents who had a good knowledge for each specific situation. In this subgroup, treatment limitation and discontinuation, double-effect treatment, and sedation for distress are the situations most practiced (91%, 95% and 86%, respectively). Deep and continuous sedation maintained until death was already witnessed by only 70% of the respondents with a good overall knowledge of this situation. More strikingly, euthanasia and assisted suicide were already witnessed 29% and 24%, respectively, of the respondents with a good overall knowledge of these situations.

Among respondents with a good overall knowledge on euthanasia (*n* = 83, 45%) and assisted suicide (*n* = 131, 72%), 24% and 42%, respectively, were favorable to decriminalization or legalization. 12% and 38% respectively felt ready to realize the act. Regarding euthanasia, this negative opinion on decriminalization or legalization was associated with a better knowledge of this practice (*p* = 0.038, see Table 3).

**Table 3:** Opinion of French heathcare workers in hematology about assisted suicide and euthanasia

	Knowledge on assisted suicide		All		*p*-value	
	*Inaccurate*	*Accurate*				
	*N* = 55	*N* = 138				
**Favorable to decriminalization or** **legalization**	16 (29%)	56 (41%)	72 (38%)		0.3	
**Would feel ready to realize the act**					0.15	
*Yes*	13 (24%)	53 (38%)	66 (34%)			
*No*	26 (47%)	53 (38%)	79 (41%)			
*Don’t know*	16 (29%)	32 (23%)	48 (25%)			
	**Knowledge on euthanasia**		**All**		**p-value**	
	*Inaccurate*	*Accurate*				
	*N* = 100	*N* = 85				
**Favorable to decriminalization or legalization**	30 (30%)	18 (21%)	48 (26%)		**0.038**	
**Would feel ready to realize the act**					**0.019**	
*Yes*,* always*	16 (16%)	10 (12%)	26 (14%)			
*Depending on specific situation*	25 (25%)	11 (13%)	36 (19%)			
*No*,* never*	47 (47%)	59 (69%)	106 (57%)			
*Don’t know*	12 (12%)	5 (5.9%)	17 (9.2%)			
*Statistics presented: n (%)*,* Statistical tests performed: chi-square test of independence*						

### Conditions and reasons for legalizing MAID

We obtained 61 responses concerning the conditions for legalizing assisted suicide out of the 72 people in favour of legalizing it (85%), and 28 responses concerning the conditions for legalizing euthanasia out of the 49 people in favour of legalising it (57%). Regarding the conditions for legalizing euthanasia at the patient’s request and assisted suicide, a thematic analysis of the verbatim responses identified the following 3 themes and 11 sub-themes, presented in Table 4. The most frequently identified conditions for legalizing euthanasia and assisted suicide were the assurance of an informed choice (61% and 28% respectively) and the collegial decision-making (57% and 46% respectively). The other criteria were the patient’s clinical condition, such as the presence of refractory suffering (21% and 22%), or the incurability of the disease (21% and 18%), but also criteria found concerned the procedure and its means, such as prior psychological assessment (29% and 22%) or the precise framework of the procedure (legislative, based on professional recommendations, etc. (21% and 22%). The presence of a third party (such as a PC team) in the decision-making process, the training and support of professionals carrying out MAIDs, and the opinion of family and friends were rarely mentioned.

**Table 4:** Percentage of occurrence of themes and subthemes for the conditions for legalizing assisted suicide and euthanasia at the patient’s request, for people in favor of legalization

Theme	Subtheme	Euthanasia (*n* = 61, 85%)	Assisted suicide (*n* = 28, 57%)
The patient’s request	Assurance of an informed choice	37 (61%)	8 (28%)
	Repetition of the request	8 (13%)	6 (20%)
	The opinion or information of the patient’s relatives	8 (13%)	4 (15%)
The clinical condition of the patient	The incurability of the disease	13 (21%)	5 (18%)
	The presence of intractable suffering	13 (21%)	7 (22%)
	The presence of a feeling of unworthiness	2 (4%)	1 (4%)
the procedure and its means	Collegial decision-making	35 (57%)	13 (46%)
	Prior psychological assessment	17 (29%)	7 (22%)
	Training and support for professionals implementing the procedure	6 (10%)	2 (7%)
	The precise framework of the procedure (legislative, based on professional recommendations, etc.)	13 (21%)	7 (22%)
	The presence of a third party team (such as palliative care)	0 (0%)	1 (4%)

Thus, the analysis of answers concerning the conditions for legalization identified three themes, namely: the clinical condition of the patient, the patient’s request, the procedure and its means. The participants’ reasons which justify their position (favourable or unfavourable) on legalizing assisted suicide or euthanasia corresponded to these same themes, to which we have added a fourth: the conception of care. The main themes and sub-themes are shown in supplementary data S4 and S5.

### Theme 1: the patient’s request

Healthcare professionals in favor of euthanasia or assisted suicide mainly referred to respect for the patient’s request: respect for his or her wishes and choice, reflecting the ideal of free will in the face of death. In contrast, participants who are against legalization consider that it would be problematic to respect the wishes of patients, given the ambivalence of humans regarding death. *“I think that everyone should be in control of their own life and death”.*

### Theme 2: the clinical condition of the patient

Healthcare professionals that were proMAID legalization wished to avoid patients suffering as a result of the deterioration of their clinical condition, the incurability of their pathology or the cessation of specific treatments - situations that could be considered “unbearable”. The supposed benefit to the patient’s relatives of this type of ‘chosen’ death was also mentioned, considered as a ‘dignified’ way of dying. *" The right to die with dignity*,* when no treatment is possible”.*

In a consequentialist vision of care, healthcare professionals in favour of legalizing MAID wished to be “fair” to patients, offering help to those whose pathology does not allow them to request limitations or cessation of life-threatening treatment. *“In haematology*,* we also encounter refusals of care that will accelerate the pathological process towards death*,* and this is perfectly acceptable (…)”.*

### Theme 3: the procedure and its means

Some participants requested a “procedural” aspect that frames the performance of these acts following an external psychological assessment and a collegial decision. For some people, respecting the decision made by the collegial body was a sufficient reason for providingMAID. Considering that the teams involved in patient care are unable to carry out these procedures, due to a lack of expertise or time, other respondents mentioned the resort to « specialized teams » as an option. *“As far as I’m concerned*,* in onco-haematology*,* MAID is not our job.”*

### Theme 4: the conception of care

The conception of care influenced participants’ answers. Those opposed to MAIDmentioned possible therapeutic alternatives (such as stopping transfusions, deep and continuous sedation until death, double effect, comfort care, psychological support and/or PC). In their desire to provide better symptom relief and support for patients, some mentioned the need for better deployment of PC across the country. *“I think that other means could have been used to deal with his moral distress*,* such as increased psychological care*,* a support team adapted to his loss of autonomy (and which he can tolerate)*,* effective pain management*,* etc. (…)”*.

Some participants considered that “giving death” is contrary to the caring function, and particularly to the role of the physician, who is more directly involved in MAID, according to the participants’ representation. They referred to the Hippocratic Oath, which states that physicians must not take power over the life or death of others. *“We are healthcare professionals and we have been trained to heal and care. If curative treatment is no longer possible*,* our role is to accompany*,* support and relieve*,* but not to give death.”*

Others stated a position based on moral or religious principles. *“As a Catholic*,* I cannot accept any act whose sole aim is to kill the patient”.*

Some people worried about the negative repercussions that this law could have on all vulnerable people at the EOLe and on their support. *“Are some lives less worth living than others? How might patients who are losing their autonomy but not expressing a request for assisted suicide feel if they see that others*,* sometimes less degraded than themselves*,* are making such a request? (…)”*.

Others wondered who should be responsible for providing MAID: should it be carried out by an outside team or by the haematology team?

Finally, the question of the impact of MAID on carers was put forward as an argument against legalizing assisted suicide or euthanasia. Furthermore, the repercussions of euthanasia were considered as potentially more negative for carers than accompanying a patient to assisted suicide. *“I won’t be able to carry out this act*,* I find it traumatic for the healthcare professional making the decision and the person carrying it out”.*

Among people who did not take a position in favor or against MAID, the same types of arguments were found: the ability to relieve suffering, the concept of care and the medical function, and the impact on the healthcare professional. Questions about uncertainty, inadequate information on the clinical situation and the lack of expertise in the field of PC and EOL care were the main reasons for the absence of a “for or against” position, as was the need to individualize decisions. Finally, one respondent referred to the limitations of the practical conditions for carrying out such practices (lack of resources), which were “time-consuming” or “unrealistic”. *“This procedure will take time*,* and is it feasible in the context of intense daily haematology activity?”*

## Discussion

Our study shows that the knowledge of different EOL situations by haematology healthcare professionals and their lawfulness is not perfect. There is lower knowledge of situations involving double effect, euthanasia and sedation for distress. Haematology healthcare professionals have a better knowledge of LATA situations. Training in PC stood for the main driver of carers’ knowledge of complex EOL situations, as well as being a physician. Training in PC is a major issue in haematology departments, which face many complex EOL situations. More frequent collaboration with PC teams could improve hematology teams’ knowledge [14, 32]. Accurate identification of the different EOL care situations represents an essential step in offering patients high-quality PC, while strengthening their access to rights guaranteed by a changing legal framework [33].

Knowledge was associated with the opinion on assisted suicide and euthanasia: those with good knowledge of euthanasia definition were less in favour of legalization and would be less inclined to carry it out, whereas this relationship was less significant for assisted suicide.

Participants with a good knowledge of these situations had often encountered them in their clinical practice (over 90% for LATA and double effect situations and over 80% for sedation for distress). The deep and continuous sedation was only carried out by 70% of participants who recognize it, while almost one professional in four believed they had already been confronted with a situation of euthanasia or assisted suicide, even though these situations are illegal in France.

Euthanasia and assisted suicide carried out clandestinely are, by their very nature, particularly difficult to quantify, and seem to persist even in the event of legalization, as various reports in Belgium and the Netherlands have shown [23, 34]. We may question what these clinical practices really are: given the nature of EOL situations, which are emotionally intense and require regular therapeutic adaptations, they may not be interpreted and identified in the same way by the different members of the same team, or by patients and their relatives. Yet this seems to be a major challenge in terms of training and support for the teams who carry them out, in order to improve the experience and quality of care in these EOL situations.

Opinions of healthcare professionals regarding the legalization of MAID in France were diverse and well-founded. The arguments for and against legalizing MAID refered to common principles, such as beneficence (relieving suffering): a first conception attached to the principle of non-maleficence, where the relief of the patient’s suffering is limited by the prohibition on “giving death”; and a second guided by the principle of autonomy, where respect for the patient’s expressed wishes and the desire to put an end to his suffering come together. The MAID decision seemed to be strongly linked to the medical function, although the principle of collegiality, which has been present in French legislation for nearly 20 years in EOL situations, was very often evoked. The question of who performs these procedures was also not clear-cut, with some preferring the referral haematology team and others a third party team, such as PC. Finally, it is worth noting that, in the participants’ opinion, the patients’ experience of incurability and the cessation of specific treatments were considered a sufficient reason to justify MAID. This corroborates numerous qualitative studies highlighting the difficulty for haematologists in stopping specific treatments and thus destroying the patient’s hope [35–38].

Another qualitative study has explored the French oncologists’ position on euthanasia and their experiences in dealing with patients who request it [24]. In this study, most of the 24 oncologists or haematologists interviewed were opposed to legalizing euthanasia, citing concerns about the societal, medical and ethical implications of this practice. They refused physician-assisted suicide based on their ethical duty to preserve life, avoid harm and uphold the Hippocratic Oath. According to participants, patient requests for euthanasia were rare. Our study seemed to be more nuanced with regard to the opinion of healthcare professionals, who seemed less homogeneous in our survey.

In addition, another recent survey was carried out in France on the opinion of healthcare professionals regarding the legalization of MAID [39]. In this study, 1439 PC stakeholders (19% volunteers, 33% nurses and 26% physicians) took part and expressed an opinion about the legalization of MAID. 1053 (69.7%) were against the legalization of MAID. When forced to choose which option should be privileged if the law had to change, 3.7% favoured euthanasia, 10.1% favoured assisted suicide with provision of lethal drug by a professional, 27.5% favoured assisted suicide with prescription of a lethal drug and 29.5% favoured assisted suicide with provision of a lethal drug by an association. The opinion regarding legalization of MAID was statistically different depending on the participant profession (*p* < 0.001). Nurses and physicians were statistically more reticent about MAID than other professionals, and there was no relationship with age or number of years’ experience in PC. Compared with our study, PC stakeholders appeared to be more reticent than haematology professionals about changes to the legal framework. The survey did not assess participants’ level of knowledge about EOL situations.

### Strengths

To the best of our knowledge, our study was the first to have specifically questioned haematology healthcare professionals, analyzing their opinions in relation to their knowledge of the EOL situations. In this way, we were attempting to establish training priorities, as well as keys to understanding and providing support for French healthcare professionals faced with changes in the legal framework in which they work. The Ethics Committee of the French Society of Haematology will thus be able to adapt its online training courses and the themes of its training days to the results of this survey.

The multi-professional profile of our sample was a strength, in an area where it is sometimes difficult for teams to share their views. The design of our study was a mixed methodology making it possible to interpret the quantitative data using qualitative results, according to the definition of mixed studies proposed by Pluye and Vedel (Mc Gill University) [40]. This design allowed for a more in-depth interpretation of the links between training and opinion, and a better understanding of the results obtained.

### Limitations

Given the current legislative situation in France, it was relevant to gain a better understanding of clinicians’ knowledge and opinions regarding possible changes to the legal framework. In the absence of a validated tool on this subject, we designed a targeted questionnaire, based on clinical vignettes, to assess health care professionals’ knowledge of the latest EOL legislation and its application on the specific hematology context. Due to the time constraints, it was not possible to go through all the validation and reliability stages required to make it a rigorous research tool. Therefore, external validation of this survey should be treated with caution.

Our results are based on a transversal survey collecting only the opinions of participants who consented to participate in the study. Given the size of the overall target population (haematology healthcare professionals in France), this study could not claim to be representative of the whole population. Furthermore, due to the sampling method, non-respondents could not be studied or compared to respondents.

Regarding qualitative data, we observed a successive loss of responses as the survey progressed. It was also difficult to analyze very short responses with numerous contradictions, limiting the qualitative analysis. Semi-structured interviews should be performed to complete the results.

## Conclusions

French hematology healthcare professionals have lesser knowledge of situations involving double effect, euthanasia and sedation for distress than of situations involving limiting or stopping treatment. Knowledge of specific EOL situations has a different influence on opinion of legalization of MAID.

## Electronic supplementary material

Below is the link to the electronic supplementary material.

Supplementary Material 1

Supplementary Material 2

Supplementary Material 3

Supplementary Material 4

Supplementary Material 5

## Data Availability

The quantitative data used to support the findings of this study are available from CB. The qualitative data used to support the findings of this study are available from CP and AP.

## Abbreviations

SFH: Society of Haematology
MAID: medical assistance in dying
PC: Palliative Care
AFITCH-OR: French Association of Nurses working in cell therapy, hematology, oncology and radiotherapy
GFIC-GM: French Group of Stem cell transplant coordinator nurses
IQR: Interquartile range
