# Supplementary material for: Cross-referencing French hematology teams’ knowledge and perception of end-of-life situations: a national mixed-methods survey
Source: BMC Palliat Care. 2025 Jan 31;24:32. doi: 10.1186/s12904-025-01659-9 (PMC11786354; doi:10.1186/s12904-025-01659-9)
Supplement: Supplementary file 1 — Supplementary Material 1 [file 12904_2025_1659_MOESM1_ESM.docx]

**Supplementary data:**

***Table S2:*** Univariate and multivariate analyses evaluating predictors of the knowledge score

| **Variables** |  | **Knowledge score (/10)** | **Univariable analysis** | **Multivariable analysis** |
| --- | --- | --- | --- | --- |
|  |  | *Mean (SD)* | *Coefficient (95% CI)* | *Coefficient (95% CI)* |
| Sex | Female | 6.7 (2.2) | - | - |
|  | Male | 6.7 (2.4) | 0.03 (-0.50 to 0.56, p=0.911) | - |
| Age | <30 | 7.1 (2.6) | - | - |
|  | 30-50 | 6.9 (2.1) | -0.27 (-0.88 to 0.35, p=0.398) | -0.26 (-0.84 to 0.33, p=0.387) |
|  | 51-60 | 5.9 (2.6) | -0.93 (-1.72 to -0.14, p=0.021) | -0.98 (-1.72 to -0.24, p=0.010) |
|  | >60 | 6.6 (2.0) | -0.45 (-1.50 to 0.61, p=0.404) | -0.84 (-1.85 to 0.17, p=0.102) |
| Professional category | Non-physician | 6.6 (2.1) | - | - |
|  | Physician (M.D.) | 7.3 (2.9) | 0.79 (0.34 to 1.24, p=0.001) | 0.85 (0.40 to 1.29, p<0.001) |
| Specialty | Hematology | 6.7 (2.9) | - | - |
|  | Oncology | 6.0 (2.6) | -0.49 (-1.92 to 0.95, p=0.502) | - |
|  | Palliative care | 8.1 (2.9) | 0.98 (-0.34 to 2.29, p=0.144) | - |
|  | General practice | 4.3 (2.0) | -1.69 (-3.94 to 0.56, p=0.140) | - |
|  | Other | 7.0 (1.6) | 0.17 (-1.05 to 1.39, p=0.787) | - |
| Tertiary hospital | Yes | 6.7 (2.9) | - | - |
|  | No | 6.6 (2.3) | -0.10 (-0.67 to 0.47, p=0.736) | - |
| Country | France | 6.7 (2.9) | - | - |
|  | Other | 7.1 (0) | 0.31 (-1.53 to 2.16, p=0.738) | - |
| Training in Ethics/Pallative care | No | 6.4 (2.3) | - | - |
|  | Yes | 7.1 (2.1) | 0.75 (0.24 to 1.27, p=0.004) | 0.76 (0.27 to 1.26, p=0.002) |
|  |  |  |  |  |
