# Supplementary material for: Cross-referencing French hematology teams’ knowledge and perception of end-of-life situations: a national mixed-methods survey
Source: BMC Palliat Care. 2025 Jan 31;24:32. doi: 10.1186/s12904-025-01659-9 (PMC11786354; doi:10.1186/s12904-025-01659-9)
Supplement: Supplementary file 2 — Supplementary Material 2 [file 12904_2025_1659_MOESM2_ESM.docx]

***Supplementary data***

***Table S3***: Knowledge on end-of-life situations according to training in medical ethics or palliative care. *Statistics presented: median (IQR); n(%). Statistical tests performed: Kruskall-Wallis test; chi-square test, Fisher’s exact test.*

|  | **Training in medical ethics or palliative care** | | **p-value** |
| --- | --- | --- | --- |
|  | No | Yes |  |
|  | *n=131* | *n=51* |  |
| Overall knowledge |  |  | 0.027 |
| *Extensive* | 36 (27%) | 23 (45%) |  |
| *Average* | 60 (46%) | 22 (43%) |  |
| *Limited* | 35 (27%) | 6 (12%) |  |
| Identification of situations (/10) | 7.1 (5.7,8.6) | 7.1 (7.1,9.3) | 0.054 |
| Legal knowledge (/10) | 8.6 (7.1,10.0) | 10.0 (8.6,10.0) | <0.001 |
|  |  |  |  |
| Specific knowledge |  |  |  |
| *Double-effect treatment* | 53 (40%) | 30 (59%) | 0.039 |
| *Treatment limitation and discontinuation* | 118 (90%) | 50 (98%) | 0.12 |
| *Sedation for distress* | 67 (51%) | 25 (49%) | 0.9 |
| *Deep and continuous sedation maintained until death* | 93 (71%) | 50 (98%) | <0.001 |
| *Assisted suicide* | 94 72%) | 37 (73%) | 0.9 |
| *Euthanasia* | 57 (44%) | 26 (51%) | 0.5 |
