# Supplementary material for: Cross-referencing French hematology teams’ knowledge and perception of end-of-life situations: a national mixed-methods survey
Source: BMC Palliat Care. 2025 Jan 31;24:32. doi: 10.1186/s12904-025-01659-9 (PMC11786354; doi:10.1186/s12904-025-01659-9)
Supplement: Supplementary file 3 — Supplementary Material 3 [file 12904_2025_1659_MOESM3_ESM.docx]

**Supplementary data S4**

***Table S4****:* Reasons for legalising assisted suicide or not. *Significant quotations from open-ended answers.*

| **Participants in favor of assisted suicide (52 responses out of 66 in favor)** | | |
| --- | --- | --- |
| Theme 1:  The patient's situation | Presence of refractory suffering | *"Do not subject people to intolerable physical and psychological suffering when they are no longer capable of coping with it."* |
|  | Incurability | *"A patient with no further hope of improvement, no therapeutic project leading to a prolonged life expectancy, who does not wish to see himself wither away physically and psychologically."*  *"Patient doomed in the short term* |
|  | Feeling of unworthiness | *"Enabling them to die with dignity, accompanied and gently".*  *"Less suffering for loved ones too".* |
|  | Discontinuation of specific treatments | *"The right to die with dignity, when no further treatment is possible."*  *"Absence of a therapeutic project Not to do so is to force the patient to undergo slow agony."* |
| Theme 2:  The patient's request | Informed choice | *"I hope that the law will evolve so that this possibility exists (with the appropriate framework) in a legal manner in France.*  *"The patient has the right to decide whether or not to die".*  *"Patient's free will* |
| Theme 3:  The procedure | The precise framework of the procedure (legislation, professional recommendations, etc.) | *"If he wishes to end his life in order to retain his dignity and suffer less, it might as well be done in a medical setting, limiting the suffering of the patient and his loved ones as much as possible."*  *"I think it's a practice that's already being carried out, but in a 'hidden' way".* |
|  | Training and support for professionals who implement the practice | *"The act of 'euthanasia' can be more difficult for caregivers"* |
|  | Collegial decision-making | *"In order to apply the collegial measures thus decided!* |
|  | Prior psychological assessment | *"If the law and all the conditions are met, reflection period, multidisciplinary assessment, elimination of a depressive syndrome accessible to treatment, pain assessment and agreement or audit of relatives."* |
| Theme 4:  Concept of care | Impact on caregivers | *"The act of 'euthanasia' can be more difficult for caregivers"* |
|  | Ability to respond with alternatives | *"In haematology, we also encounter refusals of care that accelerate the pathological process towards death, and this is perfectly acceptable...".* |
|  | The caregiver's personal position | *"No ethical opposition, but a need for prior collegial discussion".*  *"I see no reason to oppose it except religious."* |
|  | Conception of the medical function | *"It seems natural to me that hematologists should see their patients through to the end, whether the outcome is a happy one or not. We must assume our role and be able to put an end to the suffering of our incurable patients in conditions of sufficient safety and dignity."* |
| **Participants unfavorable to assisted suicide (61 responses out of 78 unfavorable)** | | |
| Theme 1:  The patient's situation | Presence of "refractory" suffering? | *"All means have not been implemented to relieve this patient here."*  *"The existential devaluation and relationship difficulties with his daughter should first be soothed".* |
|  | Feeling of unworthiness | *"Has there been any discussion of its palliative phase, taking into account the bereavement of a probably rewarding professional activity?"*  *"Human dignity is intrinsic to the human person and independent of ability, ethnicity, ect..."* |
| Theme 2:  The patient's request | Informed choice? | *"It also raises the question of how to help people who attempt suicide, because if, in the case of suicide, we judge that they are psychologically incapable of deciding for themselves, what freedom do people have when they express extreme physical or moral suffering?*  *"It's very complicated to decide on someone's death, to sort out the patient's request. Does he really want to die?"* |
|  | Repeated requests | *"How can we be sure that the patient won't have changed his mind in 15 days? With different care? Another team?"* |
| Theme 3:  The procedure | The precise framework of the procedure (legislation, professional recommendations, etc.) | *"Risk of trivialization. Very high risk of leaving such a treatment available in the home, whether taken by someone else or, worse still, voluntarily given to someone else".* |
|  | Psychological assessment | *"the request for assisted suicide often conceals something else".* |
|  | The presence of a third-party team (such as palliative care) | *"I think it would be preferable for this to be done by specialist doctors (palliative care or other) and not the referring haematologist."* |
|  | Training and support for professionals who implement the practice | *"too complicated to handle psychologically*  *"procedure. ==> Time-consuming, achievable in the midst of daily activity?"* |
| Theme 4:  Concept of care | Ability to respond to suffering or to alleviate suffering through alternatives | *"An alternative solution likely to relieve suffering is possible (continuous sedation)".*  *"Other resources to relieve people, whether physical or moral".*  *"All means have not been implemented to relieve this patient here."*  *"I think other means could have been implemented to deal with his moral distress, such as increased psychological care, a support team adapted to his loss of autonomy (and which he supports), effective management of his pain... Isn't his desire to die linked to his physical and psychological suffering? Shouldn't all this be taken care of more effectively before proceeding with assisted suicide?"* |
|  | The caregiver's personal position: moral and/or ~~religious~~ principles | *"As a Catholic, I cannot accept any act whose sole purpose is to kill the patient."*  *"It's not part of my nursing values to give death".*  *"Contrary to my religious opinions. Right of withdrawal essential if contradiction such as mine "* |
|  | Conception of the medical function | *"We are caregivers and we have been trained to heal, to care. If any curative treatment is no longer possible, our role is to accompany, support and relieve, but not to give death."*  *"I swore in the Hippocratic Oath not to intentionally cause death."*  *"As far as I'm concerned, in onco-haematology, MAID is not our job "*  *"I don't see it as a doctor's role to voluntarily administer lethal treatment, even at a patient's request. Death is a natural process, and our mission is to provide the best possible support by training in palliative care.* |
|  | Impact on the care relationship | *"The challenge is to provide the best possible support to alleviate suffering by creating a relationship of trust and developing palliative care. Assisted suicide and euthanasia will go in the opposite direction, by introducing mistrust into the relationship between caregiver and patient, or with the patient's family, and putting the brakes on palliative care."* |
|  | Impact on caregivers | *"A feeling of guilt*  *"Too complicated to handle psychologically* |
