# Supplementary material for: Cross-referencing French hematology teams’ knowledge and perception of end-of-life situations: a national mixed-methods survey
Source: BMC Palliat Care. 2025 Jan 31;24:32. doi: 10.1186/s12904-025-01659-9 (PMC11786354; doi:10.1186/s12904-025-01659-9)
Supplement: Supplementary file 4 — Supplementary Material 4 [file 12904_2025_1659_MOESM4_ESM.docx]

***Supplementary data***

***Table S5****:* Reasons for legalising euthanasia or not. *Significant quotations from open-ended answers.*

| **Participants in favor of euthanasia (25 responses out of 48 in favor)** | | |
| --- | --- | --- |
| Theme 1:  The patient's situation | Refractory suffering  Incurability | *"In human terms, it's hardly acceptable to prolong a situation of physical and psychological suffering and leave a decline in progress.*  *"It may be up to us to agree to help the patient avoid further deterioration of his living conditions and a surely unpleasant end of life, and to allow him to choose to leave in a still good state if no treatment will improve his condition anyway".* |
| Theme 2:  The patient's request | Informed choice | *"I think everyone should be master of their own life and death".* |
| Theme 3:  The procedure | Psychological assessment  Collegial procedure  Informing the patient's family and friends | *"Lock psychological evaluation, lock collegial meeting, lock patient's will, opinion of relatives heard, coordination of procedure with family and patient".* |
| Theme 4:  Concept of care | Conception of the medical function | *"I think that even if the patient's quality of life has not yet deteriorated too much, in our view as caregivers, and there is still time to live, it may be our responsibility to agree to help the patient avoid a further deterioration in his living conditions and a surely unpleasant end of life, and to allow him to choose to leave in a still good condition if no treatment will improve his condition in any case".*    *"Because I believe that listening to patients and respecting their decisions when nothing else can be offered is care."* |
| **Participants opposed to euthanasia (50 responses out of 90 opposed)** | | |
| Theme 4:  Concept of care | Ability to respond with alternatives | *"Deep and continuous sedation could have relieved her of the symptoms presented, potentially accelerating her death, without these astronomical morphine injections with the sole aim of giving death."*  *"This decision is tantamount to silencing psychic suffering in this case, and therefore runs counter to 'care', whether curative or palliative. A request for euthanasia must be listened to, heard, but taken care of: what causes suffering?"* |
|  | Personal position | *"Personal refusal to give death, even indirectly, when the patient is not 'at death's door'."*  *"Religious convictions*  *"Euthanasia is objectively homicide and I refuse to practice it, even if the law were to decriminalize the act."* |
|  | Conception of the medical function | *"It's a real loss of meaning for the medical profession, and such legalization could lead me to stop practicing medicine.*  *"I'm not a doctor to do that. I'm not a doctor to do that."*  *"I don't consider that giving death is care. Contrary to my Hippocratic oath".* |
|  | Impact on caregivers | *"I won't be able to perform this act, I find it traumatic for the caregiver who makes the decision and the one who performs it."*  *"I cannot perform a medical act with the intention of causing death. I would feel a sense of personal responsibility."* |
