# Supplementary material for: Cross-referencing French hematology teams’ knowledge and perception of end-of-life situations: a national mixed-methods survey
Source: BMC Palliat Care. 2025 Jan 31;24:32. doi: 10.1186/s12904-025-01659-9 (PMC11786354; doi:10.1186/s12904-025-01659-9)
Supplement: Supplementary file 5 — Supplementary Material 5 [file 12904_2025_1659_MOESM5_ESM.docx]

**S1: SURVEY ON END-OF-LIFE CARE IN HEMATOLOGY**

In the current context of societal discussions relating to end-of-life situations (opinion 139 of the CCNE, citizens' convention organised by the Economic and Social Council at the request of the Ministry, public information meetings organised by the regional Ethical Reflection Spaces), the SFH Ethics Commission wishes to participate in the reflection of healthcare teams on this subject. The aim of this survey is to gain a better understanding of the perceptions of healthcare professionals working in haematology with regard to complex end-of-life situations. We suggest you analyse a few clinical vignettes and answer the related questions. The vignettes do not reflect all the complexity of the clinical situation, but may refer to similar clinical cases. Your participation is free and anonymous. The results will be circulated by the SFH Ethics Committee and will be used as a basis for designing webinar-type training courses for haematology carers. Thank you for your collaboration.

**Scenario 1:**

Patient 1, over 70 years old, has an aggressive chemo-resistant B lymphoma with bone involvement, liver invasion with hepatocellular failure and lysis syndrome with acute renal failure. Patient 1 had no curative therapeutic resources and was admitted to a haematology unit because of increased liver failure and worsening bone pain. An IV morphine treatment was introduced and gradually increased in order to achieve sufficient relief of his bone pain. Because of his hepatic and renal insufficiency, these increases in treatment led to increased drowsiness and worsening of his hepatic encephalopathy, possibly hastening his death. Patient 1 died within a few days on the ward, with hetero-assessment revealing a patient who was comfortable and non-algesic most of the time.

Question 1: What type of situation is this?

- Limiting and stopping treatment
- **Double effect**
- Sedation for distress
- Deep and continuous sedation maintained until death
- Euthanasia
- Assisted suicide
- I don't know
- Other :

Question 2: Do you encounter this type of situation in your department?

-yes

-no

-I don't know

Question 3: Do you agree with the team's response to this clinical situation?

- Yes

- no

-I don't know

Question 4: In your opinion, does the procedure described in this situation comply with current legislation?

- **Yes**
- No
- I don't know

**Scenario 2**:

Patient 2, over 60 years old, was admitted to the haematology department because she had fallen at home due to major asthenia associated with refractory acute leukaemia following essential thrombocythemia. She also has a history of multiple myeloma, which has been treated several times and is currently in biological relapse. Patient 2 has been transfused weekly with EC and CPA for more than a month in the outpatient department. She has been receiving exclusive transfusion support for 6 months. When she was admitted to hospital, Patient 2 repeatedly asked that no further transfusion support be provided and that her care be focused solely on her comfort and support. The nursing care and the hospital environment enabled her to regain peace of mind and receive help with everyday tasks. She developed a nocturnal fever, which was only treated with antipyretics. She died 15 days later, in her sleep, without needing any other treatment.

Question 5: What type of situation is this?

- **Limiting and stopping treatment**
- Double effect
- Sedation for distress
- Deep and continuous sedation maintained until death
- Euthanasia
- Assisted suicide
- I don't know
- Other :

Question 6: Do you encounter this type of situation in your department?

-yes

-no

-I don't know

Question 7: Do you agree with the team's response to this clinical situation?

- Yes

- no

-I don't know

Question 8: In your opinion, does the procedure described in this situation comply with current legislation?

- **Yes**
- No
- I don't know

**Scenario 3**:

Patient 3, over 40 years old, married with 3 young children, was being treated for mediastinal lymphoma refractory to all lines of therapy. A history of renal transplantation contraindicates Car T cell treatment. He is under the care of the HAH team for palliative care. At home, he presented with rapidly progressive dyspnoea and oedema of the face and right arm. Because of his discomfort, he was taken to emergency at the Regional Hospital Centre. Symptomatic treatment with morphine and IV anxiolytics initially improved his comfort, and a transfer to general medicine was organised. The following night, patient 3 presented with acute respiratory distress refractory to symptomatic treatment. The senior on-call nurse injected him with repeated boluses of midazolam to induce unconsciousness, then maintained a dose of benzodiazepine sufficient to keep him sedated. He died 2 hours later in the presence of his wife.

Question 9: What type of situation is this?

- Limiting and stopping treatment
- Double effect
- **Sedation for distress**
- Deep and continuous sedation maintained until death
- Euthanasia
- Assisted suicide
- I don't know
- Other :

Question 10: Do you encounter this type of situation in your department?

-yes

-no

-I don't know

Question 11: Do you agree with the team's response to this clinical situation?

- Yes

- no

-I don't know

Question 12: In your opinion, does the procedure described in this situation comply with current legislation?

- **Yes**
- No
- I don't know

**Scenario 4**:

Patient 4, over 20 years old, is admitted to the haematology department with superior cava syndrome as part of a refractory diffuse T lymphoma. A final project for inclusion in an early-phase protocol is being investigated, for which she has to meet the investigating doctor 2 hours away from her referral centre. Unfortunately, her respiratory condition is deteriorating daily. She had continuous dyspnoea and was constantly sitting bent over in bed. Her laboratory work-up revealed a major lysis syndrome with progressive renal failure. Despite analgesic and anxiolytic treatments, her dyspnoea remains severe, with a constant fear of suffocating to death. The protocol inclusion project was abandoned. She asked the doctors for continuous sedation. After assessing the patient with the psychologist, the haematology care team, the referring doctor and the doctor from the mobile palliative care team met as a group to decide whether patient 4 's request was admissible. Given the poor short-term prognosis, the refractory symptoms and the patient's repeated requests, it was decided to start deep sedation with midazolam, which would be maintained until her death. It was started that very afternoon, in the presence of her parents. The patient died in the early hours of the morning.

Question 13: What type of situation is this?

- Limiting and stopping treatment
- Double effect
- Sedation for distress
- **Deep and continuous sedation maintained until death**
- Euthanasia
- Assisted suicide
- I don't know
- Other :

Question 14: Do you encounter this type of situation in your department?

-yes

-no

-I don't know

Question 15: Do you agree with the team's response to this clinical situation?

- Yes

- no

-I don't know

Question 16: In your opinion, does the procedure described in this situation comply with current legislation?

- **Yes**
- No
- I don't know

**Scenario 5**:

Patient 5, over 60 years old, a former company director, suffers from multiple myeloma that is refractory after 8 lines of treatment. Numerous bone locations and multiple pathological fractures prevent him from walking and restrict all activities of daily living. Patient 5 is in a great deal of pain and is poorly relieved by level 1 and 2 analgesics, but he is very reluctant to use opioids because he has long-standing stable renal failure. At the re-evolution consultation, patient 5 spoke of significant existential suffering, with a loss of meaning and a feeling of unworthiness linked to his loss of autonomy. He no longer went out and couldn't stand his home carer. He suffers from the "condescending" look his daughter has given him since his illness. He asks you to prescribe him a pill so that he can kill himself, because he doesn't want to live out his last weeks in this state, which you do after discussing it with the paramedical team.

Question 17: What type of situation is this?

- Limiting and stopping treatment
- Double effect
- Sedation for distress
- Deep and continuous sedation maintained until death
- Euthanasia
- **Assisted suicide**
- I don't know
- Other :

Question 18: Do you encounter this type of situation in your department?

-yes

-no

-I don't know

Question 19: Do you agree with the team's response to this clinical situation?

- Yes

- no

-I don't know

Question 20: In your opinion, does the procedure described in this situation comply with current legislation?

- Yes
- **No**
- I don't know

Question 21: Would you be in favour of legalising or decriminalising assisted suicide in this situation?

- Yes
- No
- I don't know
- If so, under what conditions?

Question 22: If this procedure were decriminalised or legalised, would you be prepared to carry it out?

- Yes
- No
- I don't know
- Could you please explain in a few words why?

**Scenario 6:**

Patient 6, over 90 years old, was admitted to your department overnight following a massive haemorrhagic stroke with subtentorial involvement, as part of an MDS under exclusive supportive care with profound thrombocytopenia. On arrival, her Glasgow score was 4, eupneic with a respiratory rate of 14/min, respiratory congestion, mottling and some myoclonus of the lower limbs. Her face appeared relaxed. She had no family and was living in a nursing home. In view of the terminal phase of the disease and the lack of certainty about her comfort, you decide to inject repeated boluses of 10mg IV morphine every 5 minutes until her respiratory rate drops and she stops breathing. Patient 6 dies quickly in the aftermath, in the presence of the nurse.

Question 23: What type of situation is this?

- Limiting and stopping treatment
- Double effect
- Sedation for distress
- Deep and continuous sedation maintained until death
- Euthanasia
- Assisted suicide
- I don't know
- **Other :**

Question 24: Do you encounter this type of situation in your department?

-yes

-no

-I don't know

Question 25: Do you agree with the team's response to this clinical situation?

- Yes

- no

-I don't know

Question 26: In your opinion, does the procedure described in this situation comply with current legislation?

- Yes
- **No**
- I don't know

Question 27: Would you be in favour of legalising or decriminalising euthanasia in this situation?

- Yes
- No
- I don't know
- If so, under what conditions?

Question 28: If this procedure were decriminalised or legalised, would you be prepared to carry it out?

- Yes
- No
- I don't know
- Could you please explain in a few words why?

**Scenario 7:**

Patient 7, over 50 years old and divorced, with an only son who committed suicide 10 years ago, is admitted to your department with febrile aplasia in the setting of refractory acute leukaemia. She is pancytopenic, transfused weekly with CPA and CE. Broad-spectrum antibiotics rapidly brought the sepsis under control. She is bothered by constant metrorrhagia, for which she is transfused with CPA almost daily. She can no longer tolerate being in hospital and considers her quality of life to be very limited. She is asking you and the paramedical team to administer a lethal product to put an end to it. She was able to meet with the liaison psychiatry team, who found no worsening of a depressive syndrome, as the patient had already been taking antidepressants since the illness was announced. Patient 7 had been monitored by the mobile palliative care team on several occasions, and refused to be transferred to a palliative care unit. After a collegial procedure with the entire care team, her referring doctor and the opinion of her GP, you administered a barbiturate combined with an IV neurotransmitter blocker at her request one morning. She died a few moments later.

Question 29: What type of situation is this?

- Limiting and stopping treatment
- Double effect
- Sedation for distress
- Deep and continuous sedation maintained until death
- **Euthanasia**
- Assisted suicide
- I don't know
- Other :

Question 30: Do you encounter this type of situation in your department?

-yes

-no

-I don't know

Question 31: Do you agree with the team's response to this clinical situation?

- Yes

- no

-I don't know

Question 32: In your opinion, does the procedure described in this situation comply with current legislation?

- Yes
- **No**
- I don't know

Question 33: Would you be in favour of legalising or decriminalising euthanasia in this situation?

- Yes
- No
- I don't know
- If so, under what conditions?

Question 34: If this procedure were decriminalised or legalised, would you be prepared to carry it out?

- Yes
- No
- I don't know
- Could you please explain in a few words why?

**Socio-demographic data**

Question 35 : Your sex : Male, Female

Question 36: Your age range :

<30 years 30-50 years, 50-60 years, >60 years

Question 37: Your profession:

PUPH, MCUPH, PH or equivalent, CCU or AH, intern, manager, nurse, psychologist, nursing assistant, social worker, other: .....................

Question 38: Your speciality:

Haematology, Oncology, Intensive care, Palliative care, General medicine, Geriatrics, other:...

Question 39: Country of practice :

France

Other:....

Question 40: Your practice institution: University Hospital, General Hospital, Mixed hospital establishment (public/private), Private clinic, other:....

Question 41: Do you have training in palliative care or ethics: yes/no
